# Supplementary material for: Changes in Health-Related Behaviours and Mental Health in a UK Public Sample during the First Set of COVID-19 Public Health Restrictions
Source: Int J Environ Res Public Health. 2022 Mar 26;19(7):3959. doi: 10.3390/ijerph19073959 (PMC8997575; doi:10.3390/ijerph19073959)
Supplement: Supplementary file 1 [file ijerph-19-03959-s001.zip › ijerph-1608629-supplementary.pdf]

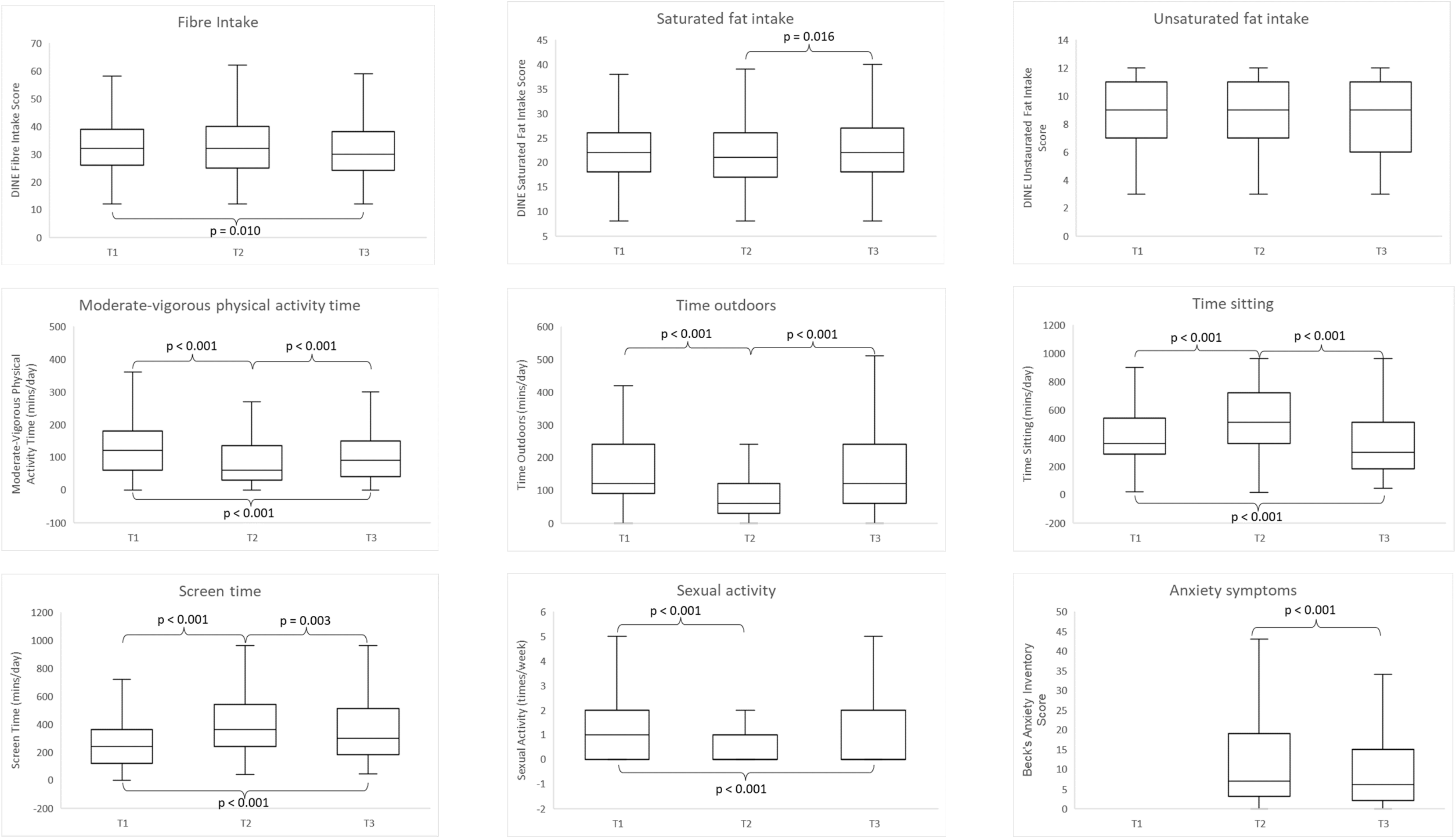
Fibre Intake
DINE Fibre Intake Score
0
10
20
30
40
50
60
70
T1
T2
T3
p = 0.010
Saturated fat intake
DINE Saturated Fat Intake Score
5
10
15
20
25
30
35
40
45
p = 0.016
T1
T2
T3
Unsaturated fat intake
DINE Unsaturated Fat Intake Score
0
2
4
6
8
10
12
14
T1
T2
T3
Moderate-vigorous physical activity time
Moderate-Vigorous Physical Activity Time (mins/day)
-100
0
100
200
300
400
500
p < 0.001
p < 0.001
p < 0.001
T1
T2
T3
Time outdoors
Time Outdoors (mins/day)
0
100
200
300
400
500
600
p < 0.001
p < 0.001
T1
T2
T3
Time sitting
Time Sitting (mins/day)
-200
0
200
400
600
800
1000
1200
p < 0.001
p < 0.001
p < 0.001
T1
T2
T3
Screen time
Screen Time (mins/day)
-200
0
200
400
600
800
1000
1200
p < 0.001
p = 0.003
p < 0.001
T1
T2
T3
Sexual activity
Sexual Activity (times/week)
-2
-1
0
1
2
3
4
5
6
p < 0.001
p < 0.001
T1
T2
T3
Anxiety symptoms
Beck's Anxiety Inventory Score
0
5
10
15
20
25
30
35
40
45
50
p < 0.001
T1
T2
T3

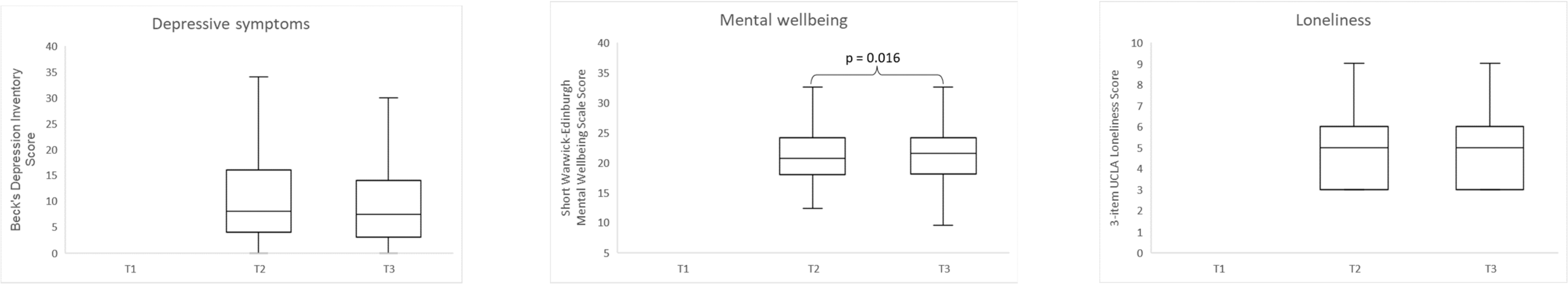

*Only significant differences ($p<0.017$ after Bonferroni adjustment for multiple comparisons) are highlighted.

Figure S1. Box and whisker plot tiles displaying changes * in lifestyle behaviours and health outcomes at different timepoints during the first set of COVID-19 public health restrictions.

Table S1. Changes in DINE fibre categories at different timepoints during the first set of COVID-19 public health restrictions.

	Fibre category at T2			Count, n	X^2	p value
**Fibre category at T1**	Low	Medium	High			
Low	92	26	2	296	5.48	p=0.140
Medium	25	62	23			
High	6	12	48			
	**Fibre category at T3**			**Count, n**	**X^2**	**p value**
**Fibre category at T1**	Low	Medium	High			
Low	95	23	2	296	6.13	p=0.105
Medium	39	56	15			
High	5	20	41			
	**Fibre category at T3**					
**Fibre category at T2**	Low	Medium	High			
Low	89	31	3	296	6.97	p=0.073
Medium	38	44	18			
High	12	24	37			

Abbreviations: T1 = timepoint 1; T2 = timepoint 2; T3 = timepoint 3

* = Significant difference (p<0.017 after Bonferroni adjustment)

Table S2. Changes in DINE saturated fat categories at different timepoints during the first set of COVID-19 public health restrictions.

	**Saturated fat category at T2**			**Count, n**	X^2	**p value**
**Saturated fat category at T1**	Low	Medium	High			
Low	232	13	1	296	4.60	p=0.203
Medium	24	22	1			
High	0	2	1			
	**Saturated fat category at T3**					
**Saturated fat category at T1**	Low	Medium	High			
Low	226	18	2	296	4.46	p=0.216
Medium	26	20	1			
High	0	3	0			
	**Saturated fat category at T3**					
**Saturated fat category at T2**	Low	Medium	High			
Low	230	23	3	296	1.41	p=0.703
Medium	20	17	0			
High	2	1	0			

Abbreviations: T1 = timepoint 1; T2 = timepoint 2; T3 = timepoint 3

* = Significant difference (p<0.017 after Bonferroni adjustment)

Table S3. Changes in DINE unsaturated fat categories at different timepoints during the first set of COVID-19 public health restrictions.

	**Unsaturated fat category at T2**			**Count, n**	**X^2**	**p value**
**Unsaturated fat category at T1**	Low	Medium	High			
Low	17	1	0	296	2.71	p=0.258
Medium	5	145	12			
High	0	13	103			
	**Unsaturated fat category at T3**					
**Unsaturated fat category at T1**	Low	Medium	High			
Low	6	11	1	296	2.83	p=0.419
Medium	18	113	31			
High	3	34	79			
	**Unsaturated fat category at T3**					
**Unsaturated fat category at T2**	Low	Medium	High			
Low	9	11	2	296	0.95	p=0.814
Medium	15	111	33			
High	3	36	76			

Abbreviations: T2 = timepoint 2; T3 = timepoint 3

* = Significant difference (p<0.017 after Bonferroni adjustment)

Table S4. Changes in compliance with MVPA guidelines at different timepoints during the first set of COVID-19 public health restrictions.

	**Meeting MVPA guidelines at T2**		**Count, n**	**X^2**	**p value**
**Meeting MVPA guidelines at T1**	Yes	No			
Yes	218	53	288	35.86	p<0.001*
No	6	11			
	**Meeting MVPA guidelines at T3**		**Count, n**	**X^2**	**p value**
**Meeting MVPA guidelines at T1**	Yes	No			
Yes	228	43	288	19.32	p<0.001*
No	10	7			
	**Meeting MVPA guidelines at T3**		**Count, n**	**X^2**	**p value**
**Meeting MVPA guidelines at T2**	Yes	No			
Yes	200	24	288	2.73	p=0.099
No	38	26			

Abbreviations: MVPA = moderate-vigorous physical activity; T1 = timepoint 1; T2 = timepoint 2; T3 = timepoint 3

* = Significant difference (p<0.017 after Bonferroni adjustment)

Table S5. Changes in compliance with sitting and screen time thresholds at different timepoints during the first set of COVID-19 public health restrictions.

	**≥480 minutes/day sitting time at T2**		**Count, n**	**X^2**	**p value**
**≥480 minutes/day sitting time at T1**	Yes	No			
Yes	104	13	280	45.10	p<0.001*
No	76	88			
	**≥480 minutes/day sitting time at T3**		**Count, n**	**X^2**	**p value**
**≥480 minutes/day sitting time at T1**	Yes	No			
Yes	86	29	284	8.48	p=0.004*
No	57	108			
	**≥480 minutes/day sitting time at T3**		**Count, n**	**X^2**	**p value**
**≥480 minutes/day sitting time at T2**	Yes	No			
Yes	122	60	284	20.25	p<0.001*
No	19	83			
	**≥480 minutes/day screen time at T2**		**Count, n**	**X^2**	**p value**
**≥480 minutes/day screen time at T1**	Yes	No			
Yes	53	5	293	37.12	p<0.001*
No	52	183			

	**≥480 minutes/day screen time at T3**		**Count, n**	**X^2**	**p value**
**≥480 minutes/day screen time at T1**	Yes	No			
Yes	41	17	294	19.01	p<0.001*
No	55	181			
	**≥480 minutes/day screen time at T3**		**Count, n**	**X^2**	**p value**
**≥480 minutes/day screen time at T2**	Yes	No			
Yes	67	38	293	0.955	p=0.328
No	29	159			

Abbreviations: T1 = timepoint 1; T2 = timepoint 2; T3 = timepoint 3

* = Significant difference (p<0.017 after Bonferroni adjustment)

Table S6. Changes in categorical mental health outcomes between T2 and T3 during the first set of COVID-19 public health restrictions.

	**BAI category at T3**		**Count, n**	**X^2**	**p value**
**BAI category at T2**	Mild	Moderate-severe			
Mild	196	13	296	5.95	p=0.015*
Moderate-severe	30	57			
	**BDI category at T3**		**Count, n**	**X^2**	**p value**
**BDI category at T2**	Mild	Moderate-severe			
Mild	233	15	296	0.31	p=0.860
Moderate-severe	17	31			
	**SWEMWBS category at T3**		**Count, n**	**X^2**	**p value**
**SWEMWBS category at T2**	Low	Medium-high			
Low	10	11	296	0.00	p=1.000
Medium-high	10	265			

Abbreviations: BAI = Beck’s Anxiety Inventory; BDI = Beck’s Depression Inventory; SWEMWBS = Short Warwick–Edinburgh Mental Wellbeing Scale; T2 = timepoint 2; T3 = timepoint 3

* = Significant difference ($p<0.05$)
